# Changes in the *Fusarium* Head Blight Complex of Malting Barley in a Three-Year Field Experiment in Italy

**DOI:** 10.3390/toxins9040120

**Published:** 2017-03-29

**Authors:** Giovanni Beccari, Antonio Prodi, Francesco Tini, Umberto Bonciarelli, Andrea Onofri, Souheib Oueslati, Marwa Limayma, Lorenzo Covarelli

**Affiliations:** 1 Department of Agricultural, Food and Environmental Sciences, University of Perugia, Borgo XX Giugno 74, 06121 Perugia, Italy; giovanni.beccari@progetti.unipg.it (G.B.); francesco.tini@studenti.unipg.it (F.T.); umberto.bonciarelli@unipg.it (U.B.); andrea.onofri@unipg.it (A.O.); marwa.limayma@gmail.com (M.L.); 2 Department of Agricultural Sciences, Alma Mater Studiorum University of Bologna, Viale G. Fanin 44, 40127 Bologna, Italy; antonio.prodi@unibo.it; 3 Bioengineering Department, Université Libre de Tunis, 30 Av. Kheireddine Pacha, 1002 Tunis, Tunisia; souheibo@yahoo.fr

**Keywords:** *Fusarium*, barley, mycotoxins, DON, T-2 toxin, FHB complex

## Abstract

In this study, conducted for three years on eleven malting barley varieties cultivated in central Italy, the incidence of different mycotoxigenic fungal genera, the identification of the *Fusarium* species associated with the *Fusarium* Head Blight (FHB) complex, and kernels contamination with deoxynivalenol (DON) and T-2 mycotoxins were determined. The influence of climatic conditions on *Fusarium* infections and FHB complex composition was also investigated. *Fusarium* species were always present in the three years and the high average and maximum temperatures during anthesis mainly favored their occurrence. The FHB complex was subject to changes during the three years and the main causal agents were *F. poae*, *F. avenaceum*, *F. tricinctum* and *F. graminearum*, which, even if constantly present, never represented the principal FHB agent. The relative incidence of *Fusarium* species changed because of climatic conditions occurring during the seasons. The FHB complex was composed of many different *Fusarium* species and some of them were associated with a specific variety and/or with specific weather parameters, indicating that the interaction between a certain plant genotype and climatic conditions may influence the presence of *Fusarium* spp. causing infections. With regard to mycotoxin contamination, T-2 toxin, in some cases, was found in kernels at levels that exceeded EU recommended values.

## 1. Introduction

With a global production of above 145 million tons and a cultivated surface of 50 million hectares in 2014 [1], barley (*Hordeum vulgare* L.) is one of the most important cereal crops in the world. Barley grains are extensively used for malt production, representing 90% of total malt destined to the beer industry [2]. In 2014, the world beer production obtained by malting barley was estimated in about 180 million tons, more than one million of which produced in Italy [1].

Many microorganisms may have a negative impact on barley cultivation, malting production and brewing processes [3]. In particular, fungi belonging to the *Fusarium* genus, infecting barley heads, represent unwanted microorganisms for the quality of final malt and beer [4,5,6,7], as well as for beer consumers’ health, due to mycotoxin contamination related to their presence [8].

In fact, *Fusarium* spp. are able to biosynthesize different types of mycotoxins that are considered a major safety concern in the malting and brewing industry, due to their negative properties, which are potentially transferable from barley grains to malt and beer [9]. To date, there is no regulation for *Fusarium* mycotoxin levels in beer, but maximum levels have been fixed for some mycotoxins in raw materials that are used for the production of this commodity [10]. However, a recent study assessing the exposure of the European population to mycotoxins through beer consumption showed that, even if no toxicological concern was associated to mycotoxin exposure for average beer consumers, the contribution of this commodity to the daily intake is not negligible for heavy beer-drinkers [11].

*Fusarium* species are able to cause *Fusarium* head blight (FHB), one of the most important diseases of barley, wheat and other cereals. At least 15 *Fusarium* species have been reported to infect barley and wheat grains but just four of them are the most frequently associated with FHB of small-grain cereals in Europe: *Fusarium graminearum* Schwabe, *Fusarium culmorum* (W. G. Smith) Sacc., *Fusarium aveanaceum* (Fr.) Sacc. and *Fusarium poae* (Peck) Woll. [12,13]. In Italy, surveys conducted during the last fifteen years, in particular on wheat, showed that *F. graminearum sensu strictu* (previous lineage 7; hereafter referred as *F. graminearum*) represents the main FHB agent and that *F. avenaceum*, *F. poae* and *F. culmorum* are prominent members of the complex [14]. However, the spectrum of *Fusarium* spp. involved in the FHB disease may vary at regional level depending on weather conditions occurring especially during plant anthesis. Moreover, climatic conditions occurring during the summer season that precedes crop sowing may influence FHB complex composition because of the impact of high temperatures on the estivation of *Fusarium* spp. inoculum on crop residues [15]. In addition, winter conditions seem also to have an influence on the FHB complex composition, due to the impact of low temperatures and of the length of below 0 °C periods on overwintering *Fusarium* species [16]. At present, the most effective way for controlling FHB in wheat is mainly represented by agronomic means and by the use of chemical fungicides [17] but the latter is not a common practice in many barley cultivations areas or under organic farming management, where the development and commercialization of less susceptible varieties to this disease would be highly desirable. FHB complexity may lead to a constant mycotoxin contamination risk because different species with different mycotoxigenic profiles, based on their climatic demand, could infect, singularly or in association, barley or wheat heads throughout the growing seasons thus causing mycotoxin co-occurrence [18,19,20,21,22]. Considering that all barley components play an essential role in achieving a superior malt quality and that the presence of *Fusarium* species has a negative impact on the malting process, it is very important to investigate the relative composition of FHB casual agents and kernel mycotoxin contamination of different malting barley varieties in different climatic areas.

Therefore, the aims of the present three-year study, conducted on several malting barley varieties in field experiments in Central Italy were to: (1) identify and assess the incidence of the mycotoxigenic fungal genera infecting malting barley grains during the investigated years in relation to the climatic conditions recorded during summer (inoculum estivation), winter (inoculum overwintering) and spring (anthesis time); (2) identify and assess the incidence of the *Fusarium* species associated with the FHB complex, isolated from barley grains, with regard to climatic conditions as well as to their distribution in the investigated varieties; and (3) determine grain contamination levels in the malting barley varieties of deoxynivalenol (DON) and T-2 mycotoxins.

## 2. Results

### 2.1. Presence of Mycotoxigenic Fungal Microorganisms in Malting Barley Grains in Relation to Climatic Conditions

**Figure 1:**
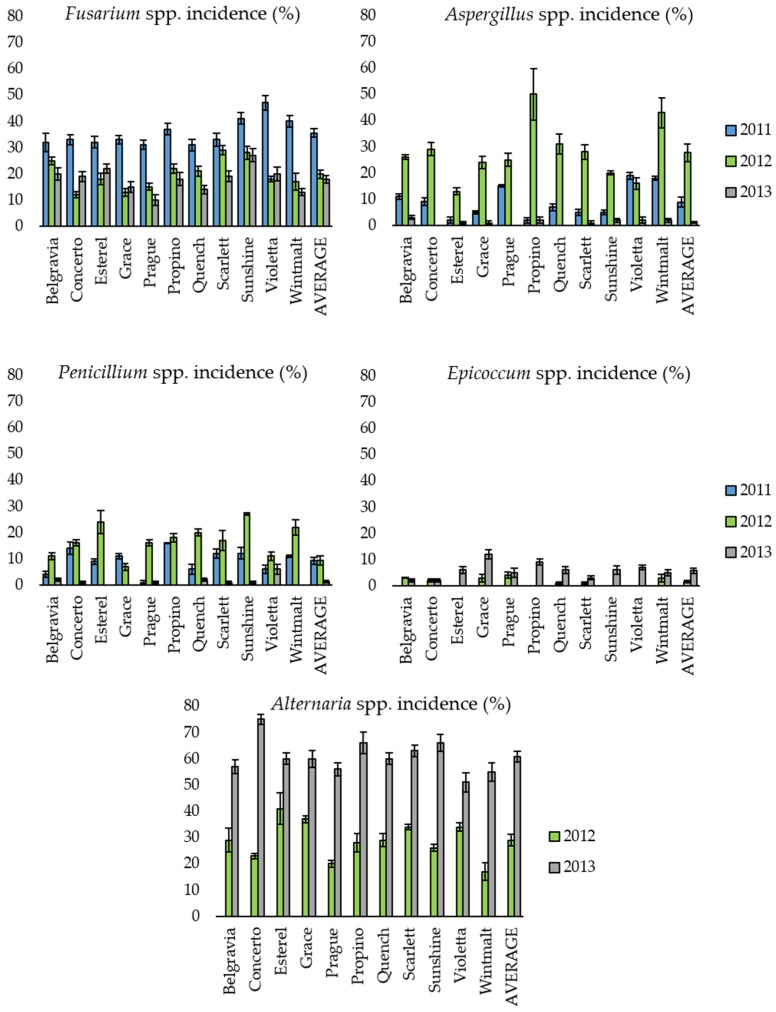
Incidence (%) of the different fungal genera isolated from the malting barley varieties in the three experimental years. Columns represent the average (± Standard Error) of four replicates per each variety. “AVERAGE” columns represent the average (± Standard Error) of the different fungal genera isolated from all analyzed varieties. In 2011, *Alternaria* spp. incidence was not determined.

A combination of visual and stereomicroscope observations allowed identification of the fungal genera infecting the analyzed malting barley samples. The mycotoxigenic genera *Alternaria*, *Fusarium*, *Aspergillus* and *Penicillium* in addition to *Epicoccum* were present with different incidences across the three growing seasons (Figure 1, Table S1).

The “year” effect was always significant (Table S1) and explained the highest proportion of data variability (0.41, 0.58, 0.56, 0.24 and 0.84, respectively, for *Fusarium* spp., *Aspergillus* spp., *Penicillium* spp.*, Epicoccum* spp. and *Alternaria* spp*.*). The “variety” effect explained a proportion of total data variability ranging from 0.01 to 0.1, depending on the fungal genus, and it was significant only in the case of *Fusarium* spp. and *Epicoccum* spp. The “variety × year” interaction was significant in almost all cases; indeed, *Fusarium* spp. was found in all years, while the other genera showed year-to-year fluctuations. For example, *Aspergillus* was particularly abundant in 2012, while it was scarcely present in 2013 (Figure 1, Table S1).

**Figure 2:**
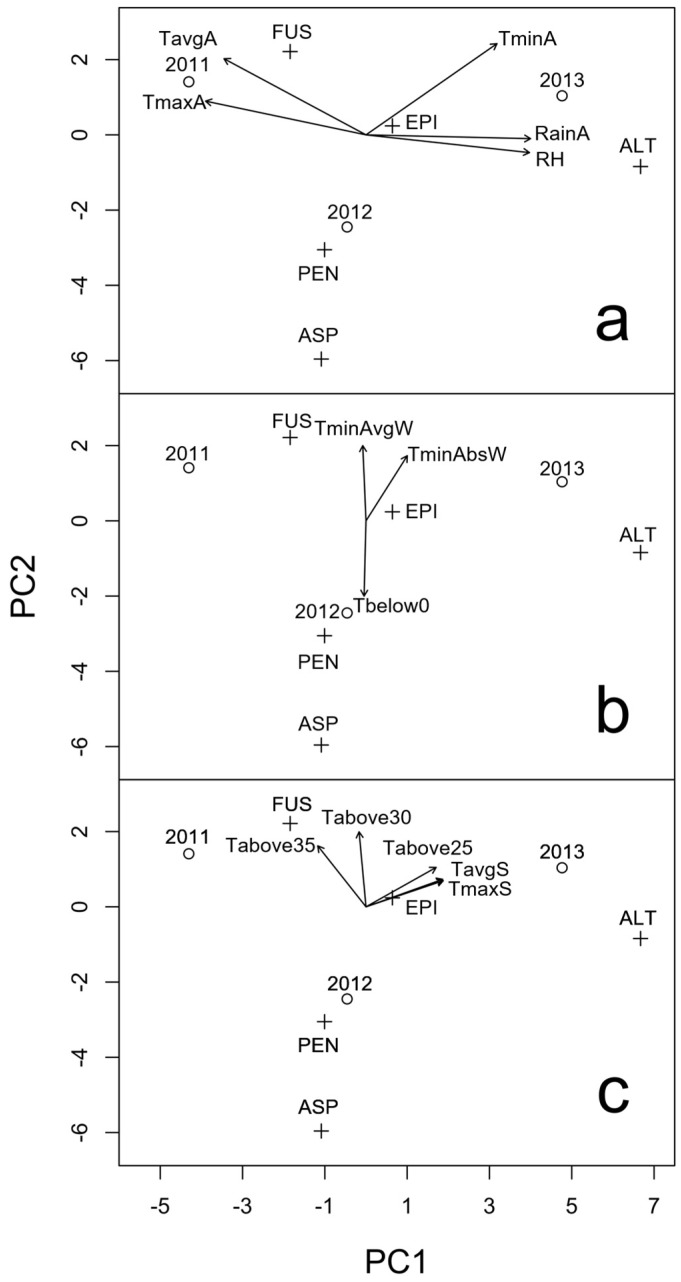
Principal Component Analysis biplot (scaling 1) of the incidence (%) of different fungal genera isolated from the malting barley varieties in the three experimental years. The arrows represent a posteriori projections of environmental variables measured: from two weeks before to two weeks after the average anthesis date (**a**); during the December–February trimester (**b**); and during the June–August trimester preceding the cultivation season (**c**). FUS, *Fusarium* spp.; EPI, *Epicoccum* spp., ALT, *Alternaria* spp.; ASP, *Aspergillus* spp.; PEN, *Penicillium* spp. See Table 2 for codes of meteorological variables. The symbols represent the exact positioning of years (o) and species (+).

In order to understand the relationship between the environment and the presence of mycotoxigenic fungal microorganisms, we considered the climatic data recorded in 2010–2013 during inoculum estivation time (June–August summer trimester before crop sowing), during inoculum overwintering time (December–February winter trimester during crop growth) and during anthesis (Table 1). These variables were projected on the ordination space, as shown in Figure 2a–c. The quality of representation on the biplot was always perfect (almost 100% of explained variability).

The year 2011 was characterized by higher average and maximum temperatures (positive values on PC1), with lower humidity and rainfall levels during anthesis (negative values on PC1) in comparison to the other years. With an incidence of 36% and negative values on the PC1, *Fusarium* was particularly present (Figure 2a). This growing season was not characterized by severe winter conditions (Figure 2b) and it was preceded by a summer period with a high number of days with temperatures above 30 °C and 35 °C (Figure 2c).

The year 2012 was characterized by negative values on PC2, with average weather conditions during anthesis. No prevalent fungal genera were recovered. *Alternaria* (29%) was slightly more represented, followed by *Aspergillus* (27%), *Fusarium* (20%), *Penicillium* (18%) and *Epicoccum* (2%). In this year, *Aspergillus* and *Penicillium* showed a higher incidence with respect to the overall mean of the three experiments (Figure 2a). In comparison to the other years, 2012 was characterized by a higher number of days with temperature below 0 °C (55 days) during winter (Figure 2b) and by a lower number of days with temperature above 30 °C and 35 °C during the previous summer (Figure 2c).

The year 2013 was characterized by a high rainfall and humidity level and high minimum temperatures during anthesis, possibly due to high cloud cover in the night. In these conditions, the genus *Alternaria* was the most prevalent one (61%) followed by *Fusarium* (18%) (positive score on PC2), *Epicoccum* (6%), and *Aspergillus* and *Penicillium* (1%) (Figure 2a). The previous summer was characterized by a higher maximum and average temperatures (Figure 2c) with respect to the other years, that seemed to have slightly negatively influenced *Fusarium* presence.

**Figure 3:**
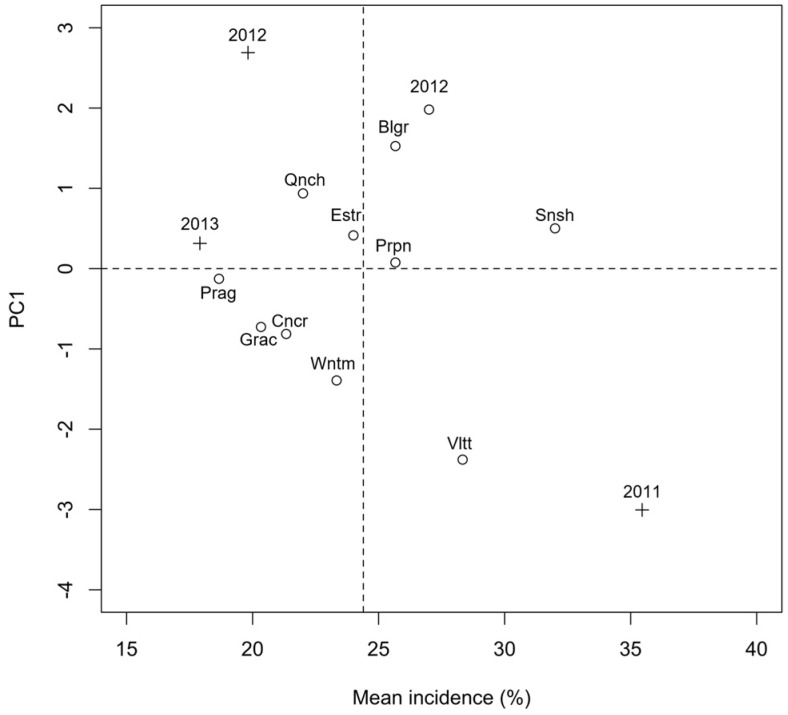
AMMI-1 biplot for the incidence (%) of *Fusarium* spp. on the grains of malting barley varieties cultivated during 2011, 2012 and 2013. Scrl, Scarlett; Blgr, Belgravia; Snsh, Sunshine; Prpn, Propino; Estr, Esterel; Qnch, Quench; Prg, Prague; Wntm, Wintmalt; Grac, Grace; Vltt, Violetta; Cncr, Concerto. The symbols represent the exact positioning of varieties (o) and years (+).

It may be interesting to more closely consider the interaction effects between years and varieties for *Fusarium* spp. To this aim, AMMI analysis is commonly used in genotype experiments to obtain a clear picture of the varieties, which showed the highest variations across the years. The AMMI-1 graph (Figure 3) shows the average incidence of the genus *Fusarium* in the 11 varieties along the *x*-axis, while the “incidence × environment” interactions are reported along the *y*-axis. In order to interpret this biplot, it should be considered that varieties on the right side of the graph showed a high average incidence across years, while varieties on the left side showed low average incidence. Furthermore, varieties positioned near to the *x*-axis (low score on the *y*-axis) did not remarkably interact with the environment and, therefore showed a fairly constant incidence across the years. Otherwise, varieties positioned away from the *x*-axis (high negative/positive scores on the *y*-axis) showed a high interaction with years and showed high incidence only in the years which lay on their close vicinity in the graph (high *y*-score of the same sign). For example, the variety “Sunshine”, in our experimental conditions, showed the highest average *Fusarium* incidence and it was above the average in all years. The variety “Violetta” was particularly attacked in 2011, while “Scarlett” and “Belgravia” were the most attacked in 2012. On the other hand, “Prague”, “Grace” and “Concerto” showed the lowest average incidence even if these latter two varieties were slightly more attacked than “Prague” in 2011.

### 2.2. Identification of *Fusarium* spp. Isolated from the Kernels, Chemotype Characterization and Their Association with Malting Barley Varieties

Following fungal isolation from barley kernels, a total of 138 *Fusarium* strains were obtained in the whole study (46 in 2011, 33 in 2012 and 59 in 2013). Species identification was performed by species-specific PCR assays or *Translation Elongation Factor 1α* (*TEF1α*) sequencing. The year effect was highly significant (chi-square = 81.2; *p*-level = 2.9 × 10^−13^).

**Figure 4:**
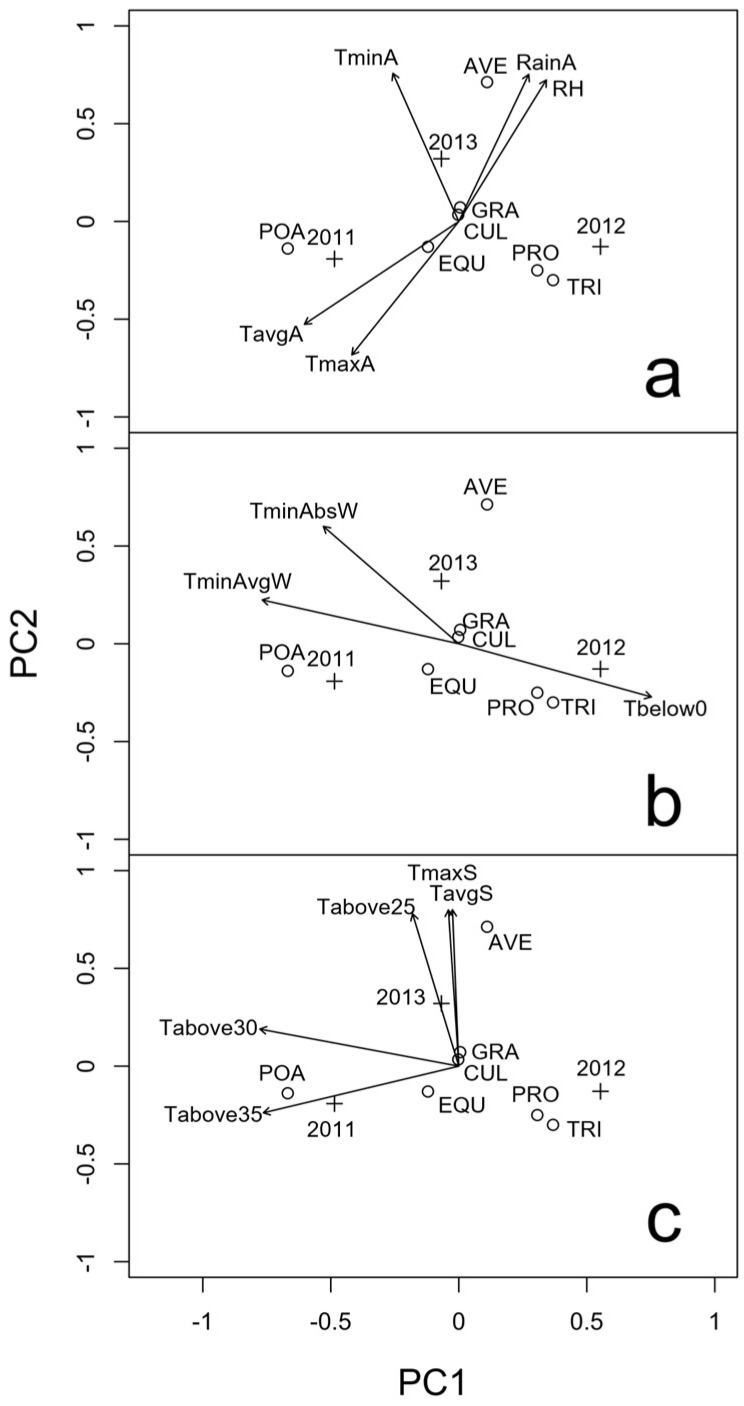
Principal Component Analysis biplot (scaling 1) of the incidence (%) of *Fusarium* spp. in the malting barley varieties as identified by PCR assays during 2011, 2012 and 2013. The arrows represent a posteriori projections of environmental variables, measured: from two weeks before to two weeks after the average anthesis date (**a**); during the December–February trimester (**b**); and during the June–August trimester preceding the cultivation season (c). AVE, *Fusarium avenaceum*; POA, *Fusarium poae*; GRA, *Fusarium graminearum*; CUL, *Fusarium culmorum*; EQU, *Fusarium incarnatum-equiseti* species complex; PRO, *Fusarium proliferatum*; TRI, *Fusarium tricinctum*. See Table 2 for environmental variables codes. The symbols represent the exact positioning of years (+) and species (o).

In 2011, *F. poae* was the most frequent species, followed by species of the *Fusarium incarnatum-equiseti* species complex (FIESC), *F. graminearum* and *F. avenaceum* (Table 2). All *F. graminearum* strains belonged to the 15-acetyl-deoxynivlenol (15ADON) chemotype (Table S2). PCA shows that *F. poae* was probably favored by high temperatures during anthesis (Figure 4a).

In 2012, a year with average climatic condition during anthesis, but with a winter characterized by a higher number of days with temperature below 0 °C, the FHB complex was characterized by a drastic change of its composition (Table 2 and Figure 4a,b). *Fusarium tricinctum* (Corda) Sacc. (36%) and *Fusarium proliferatum* (Matsush.) Nirenberg (30%) resulted to be the main species. *F. graminearum* was found at a similar level than that observed in 2011, while *F. avenaceum* incidence increased in comparison to 2011. *F. poae*, the most frequent species in the previous year, was present with a very low incidence. All *F. graminearum* strains belonged to the nivalenol (NIV) chemotype (Table S2).

In 2013, *F. avenaceum* was the most frequent species followed by *F. poae*, *F. graminearum*, FIESC and, for the first time also *F. culmorum* was recovered from the grains (Table 3). The presence of *F. avenaceum* seemed to be favored by high rainfall and humidity levels in association with low temperatures during anthesis, which characterized this year (2013). The hot climatic conditions (in terms of maximum and average temperatures) that occurred during the preceding summer did not seem to have a negative impact on *F. avenaceum* inoculum estivation (Figure 4c). Six *F. graminearum* strains belonged to the 15ADON chemotype, two to the 3-acetyl-deoxynivlenol (3ADON) chemotype and no strains belonged to the NIV chemotype (Table S2).

Considering the distribution of the single *Fusarium* spp. causing the FHB complex on the different malting barley varieties evaluated during the present study, it was observed that varieties were associated to a different number of *Fusarium* spp. (Table S3). Indeed, varieties and species composition were not independent (chi-square = 92.18; *p*-level = 0.0002). For example, the variety “Grace” was mainly infected by *F. poae*, while “Belgravia” was infected mainly by *F. poae* (83%) and, to a lesser extent, by *F. graminearum* (17%). Varieties “Esterel” and “Sunshine” were infected by an above average level of *F. avenaceum* (63% and 58%, respectively), followed by *F. graminearum* (37% and 14%, respectively) and by species of the FIESC (28% only for “Sunshine”). Varieties “Violetta” and “Propino” were found to be attacked by an above average level of *F. tricinctum* (39% and 26%, respectively) but also presented *F. poae* (28% and 40%), *F. avenaceum* (14% and 20%), *F. graminearum* (19% and 7%) and *F. culmorum* (7% only in “Propino”). Varieties “Prague”, “Scarlett” and “Wintmalt” were characterized by the simultaneous presence of *F. poae* and *F. avenaceum* (50% and 30%, 57% and 43%, 58% and 42%, respectively). “Quench” and “Concerto” were infected by several species, including *F. poae* (42% and 54%), *F. avenaceum* (11% and 18%), *F. graminearum* (17% and 18%), FIESC (3% and 2%) and *F. proliferatum* (27% only for “Quench”). In all, Quench showed the highest number of isolates with respect to all the other varieties (chi-square > 13.02; *p*-level < 0.011).

### 2.3. Occurrence of Deoxynivalenol and T-2 Toxin in the Grain

For both toxins, the effects of variety, year and “variety × year” interaction were significant (*p* < 0.0001). The presence of DON and T-2 toxin in malting barley grains of the different varieties during the three experimental years is summarized in Table 3.

In 2011, 45% of samples were contaminated with DON at an average level of 120 µg/kg and a maximum amount of 280 µg/kg. During 2012, 100% of samples were contaminated with an average DON level of 370 µg/kg and a maximum level of 480 µg/kg. Finally, in 2013, 63% of the analyzed samples were positive to DON presence with an average level of 310 µg/kg and a maximum value of 700 µg/kg. No samples were found to exceed the EU legal limit of 1250 µg/kg [10] for DON contamination.

With regard to T-2 toxin, in 2011 54% of samples were contaminated with T-2 toxin with an average level of 54 μg/kg and a maximum value of 285.9 μg/kg. In 2012, 73% of samples were positive to T-2 toxin presence with an average contamination of 126.5 μg/kg and a maximum level of 423.7 μg/kg. In 2013, all samples were contaminated with T-2 toxin, showing an average contamination level of 121.3 μg/kg and a maximum amount of 236.1 μg/kg. During the three investigated years, in four cases (12% of samples), T-2 toxin levels exceeded 200 μg/kg which is the maximum value proposed by EU recommendation 2013/165 (as the sum of T-2 and HT-2 toxins) [23]. Therefore, as HT-2 toxin was not analyzed in the present work, the incidence of samples exceeding EU recommendation was probably higher.

In 2011, 27% of samples showed the co-occurrence of detectable amounts of DON and T-2 toxin, while, 72% and 63% of samples showed the co-occurrence of these mycotoxins, respectively during 2012 and 2013. Overall, 54% of the analyzed samples during the three years showed the simultaneous presence of DON and T-2 toxin.

**Figure 5:**
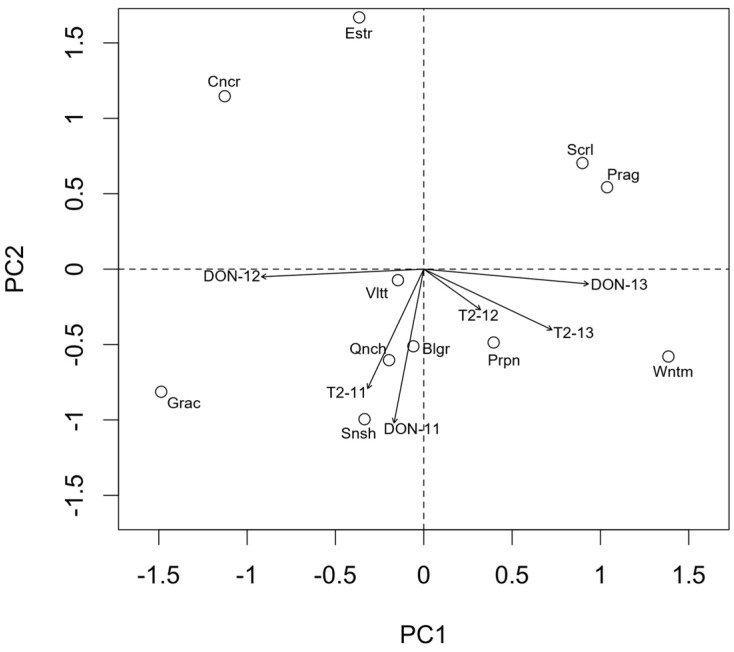
Principal Component Analysis biplot (scaling 1) for the presence of Deoxynivalenol (DON) and T-2 toxin in the 11 investigated malting barley varieties in the three experimental years. Scrl, Scarlett; Blgr, Belgravia; Snsh, Sunshine; Prpn, Propino; Estr, Esterel; Qnch, Quench; Prg, Prague; Wntm, Wintmalt; Grac, Grace; Vltt, Violetta; Cncr, Concerto; DON-11, deoxynivalenol 2011; DON-12, deoxynivalenol 2012; DON-13, deoxynivalenol 2013; T2-11, T2 toxin 2011; T2-12, T-2 toxin 2012; T2-13, T2 toxin 2013.

Considering the contamination distribution in the three years, all the varieties showed the presence of DON and/or T-2 mycotoxins (Figure 5).

Varieties “Concerto” and “Esterel” showed the lowest average DON and T-2 toxin levels, with the presence of DON only in 2012 and of T-2 toxin in 2012 (“Concerto”) and 2013 (Figure 5). “Scarlett” and “Prague” showed low DON levels in 2011 and 2012 but they were above mean contamination levels in 2013. These two varieties showed above average T-2 toxin contamination levels in 2011 and 2012 (“Prague”) and in 2013 (“Scarlett”). “Grace” was above the average contamination level for DON in 2011 and 2012 and for T-2 toxin in 2011. “Belgravia” was above average for DON only in 2012 while it was above the average for T-2 toxin in all investigated years. “Sunshine” and “Quench” were above average for DON in 2011, 2012 (“Quench”) and 2013 while in 2011 and 2012 (“Quench”) for T-2 toxin. “Violetta” was above the average for DON in all analyzed years while it was always below the average contamination level for T-2 toxin. “Wintmalt” was above the DON average level in 2011 and 2013 and above the T-2 toxin average level in 2012 and 2013. Finally, “Propino” in 2011 was below the DON average level but above the T-2 average level while it was above the average level of the analyzed mycotoxins in 2013 (Figure 5).

## 3. Discussion

*Alternaria*, *Fusarium*, *Aspergillus* and *Penicillium* were the most represented mycotoxigenic fungal genera infecting the grains of the investigated malting barley varieties evaluated in the present study. This community is very similar to that detected on malting barley in the same geographic area [24] as well as in other world areas such as Argentina [25], Denmark [26], Slovakia [27] and Spain [28]. It is interesting to underline that the most common mycoflora associated to malting barley kernels in the examined area, as well as in other cultivation areas, is mainly composed of important mycotoxigenic microorganisms, which may lead to multiple mycotoxin contaminations of this commodity.

Among the above-mentioned fungal genera, which were recovered during the present study, we focused our attention on the genus *Fusarium*, as it includes several important pathogenic species to barley that are also capable of mycotoxin production. Head infections by *Fusarium* spp. are notoriously favored by moist and warm conditions during anthesis [29,30,31,32], being these two parameters both essential to allow fungal infection development [33,34,35]. However, even if data recorded on wheat might also be useful to explain FHB dynamics in barley, macro- and micro-morphological differences in the head structure between the two cereal species and among barley varieties could influence the interaction between climatic parameters and FHB severity. In this study, the year 2011 seemed to be more favorable to *Fusarium* infections with respect to the others, being characterized by high average and maximum temperatures during anthesis, thus confirming the importance of this climatic variable during this phenological stage also for barley infections.

According to PCA, the occurrence of *Fusarium* spp. seemed to be negatively affected by the high number of days with temperatures below 0 °C, probably due to an adverse effect of low temperatures on the overwintering inoculum of *Fusarium* spp. [16]. In addition, high average and maximum temperatures during summer negatively affected *Fusarium* spp. occurrence. It is known, in fact, that prolonged high average temperatures during summer seem to be positively related to the rapidity of residue decomposition and, indirectly, this can negatively influence *Fusarium* spp. inoculum estivation [15].

Even if *Fusarium* spp. incidence changed throughout the considered years, all the analyzed barley varieties resulted to be naturally infected by these fungal species, with some varieties showing higher susceptibility with respect to others, which, however, varied in time. This may be due to the influence of climatic conditions interacting with genotypes of the cultivated material, thus originating differences in FHB response in the different cultivation seasons. During the three experimental years, the FHB complex in the examined area showed an evident change of its composition. Other studies conducted during the last decade in the same area of Central Italy and aiming at identifying *Fusarium* spp. causing FHB in soft and durum wheat showed a noticeable difference in species composition across years which was explained by the occurrence of different thermo-hygrometric conditions during the considered seasons. In particular, when climatic conditions were not favorable to the “main” FHB causal agents, such as *F. graminearum*, other “secondary” species, such as *F. avenaceum* and *F. poae*, increased their presence [20,21]. In the present study, the FHB complex composition during the years 2011, 2012 and 2013 was characterized by changes, which were slightly different in comparison to those detected in wheat in the same area. In fact, *F. graminearum* was never found to be the main FHB species and its incidence was constant but low during the three years, even when the season could have been advantageous for its development. Furthermore, *F. poae* and *F. avenaceum* were the main FHB causal agents respectively in 2011 and in 2013, being also the two occurring species in 2012 (*F. avenaceum*) and in 2013 (*F. poae*). In addition, *F. tricinctum* resulted to be an important species in the analyzed context, even if only in 2012, when a high number of days with temperature below 0 °C were recorded, when inoculum estivation conditions were not particularly favorable and when the typical FHB conditions in terms of humidity and temperature combination during anthesis were not present. Similarly, other studies conducted in malting barley in the same geographic area [24] and in different countries such as Denmark [26] and the United Kingdom [36], showed a considerably higher incidence of *F. avenaceum*, *F. tricinctum* and *F. poae* with respect to *F. graminearum*.

With regard to *F. avenaceum*, one of the most frequent species isolated from the analyzed samples, it is well-known that this fungus is favored by cold conditions [37,38] and, also in this study, *F. avenaceum* was advantaged by the combination of low temperatures and high humidity levels during anthesis. However, the presence of *F. avenaceum* has increased in Europe throughout the years, being isolated from infected grain over a wide range of climatic zones [39] and it is not possible to exclude it has adapted to an even wider range of climatic conditions. In fact, a recent genome analysis of this pathogen suggested an evolutionary adaptation to a diverse cosmopolitan ecology [40].

The present study also showed that some varieties were attacked by only one or two species but some others were also infected by up to five species at the same time. In fact, in the case of FHB, there is a community of species that causes the disease and the implications of these phenomena in the disease management should be considered, particularly in resistance studies [41]. The *Fusarium* community observed in this study seemed to possess different abilities to biosynthesize mycotoxins. For example, in addition to the typical type B trichothecene (DON and NIV) producers (*F. graminearum* and *F. culmorum*), this investigation showed the presence of *Fusarium* species able to biosynthesize both types of trichothecenes (*F. poae*), zearalenone (*F. graminearum*), beauvericin and enniatins (*F. poae*, *F. avenaceum* and *F. tricinctum*), moniliformin (*F. avenaceum* and *F. tricinctum*) and fumonisins (*F. proliferatum*) [42,43,44,45,46]. This highlights that the potential mycotoxin contamination risk of malting barley could be characterized by a wide range of compounds that may co-occur when more *Fusarium* spp. infect a single field sample at the same time.

A change across the years was also observed in the *F. graminearum* chemotype composition. In fact, even if the number of the strains of this species was not high, the predominant *F. graminearum* chemotype changed throughout the three years (15ADON in 2011, NIV in 2012, 15ADON in 2013), despite the experiments were conducted at the same location. In general, results seem to be in agreement with findings of Prodi et al. [47] and Covarelli et al. [20] obtained in the same area in durum and soft wheat.

With regard to mycotoxin contaminations, the study showed a generally low DON presence, while just T-2 toxin levels on its own exceed the EU recommended level [23] in 12% of samples. Nevertheless, as HT-2 toxin was not analyzed in the present work, the incidence of samples exceeding EU recommendation (sum of T-2 and HT-2 toxins) was probably higher. This is also supported by a recent investigation performed on malting barley in Italy, during which a higher HT-2 toxin content with respect to T-2 toxin was detected [24]. In addition, the presence of glucosyl derivative of HT-2 toxin could have been present as it was detected in malting barley kernels cultivated in Nothern Italy [48].

The results obtained in this study about the presence of DON and T-2 toxin in malting barley samples confirm the trend observed in Italy [24] and in other EU countries, such as The Netherlands, UK, Finland, France, Poland, Norway, Germany, and Czech Republic, in malting barley [49]. In fact, DON incidence seems to be stable over the years in most of the countries, with concentrations, which are generally below the EU legal limits for unprocessed barley [10]. Conversely, the incidence of T-2 and HT-2 toxins seems to increase over the years [48]. In fact, after oats, barley resulted to be one of the most contaminated cereals with HT-2 and T-2 toxins [50]. Malachova et al. [51] indicated that the presence of T-2 and HT-2 toxins in malting barley is increasing. These Authors hypothesized that this trend might be explained by the spread of *F. poae*, which is able to biosynthesize type A trichothecenes. Other Authors, have ascribed this phenomena to the presence of *Fusarium langsethiae* Torp & Nirenberg, another type A trichothecene producer [52], that in the last years increased its presence in Europe [53,54]. The absence of *F. langsethiae* in our investigations could be due to the isolation method used, which probably had a low efficiency for the detection of this slow growing species. However, this species, even if with a low incidence, was isolated in the same years and in the same area from other barley malting samples not included in the present experiment, showing its presence in the investigated area [55]. Further studies will be necessary to develop a method, or a combination of different methods, for the detection of as many as possible different *Fusarium* spp. in order to obtain a comprehensive overview of the FHB complex composition, also in relation to grain mycotoxin contamination.

T-2 toxin levels recorded in this study were similar to those detected through the years in barley in other countries, such as Germany [56], Spain [57], Norway [58], Lithuania [59], Poland [60] and Czech Republic [51]. However, even if T-2 toxin is receiving a particular attention in the malting barley chain, the contamination levels of this toxin generally decrease during the brewing process [51,61]. In fact, the analyses conducted on beer produced in 2010–2011 in several European countries showed that T-2 toxin was not present among the mycotoxins recovered in the final product [8].

## 4. Conclusions

In conclusion, the present work shows that the malting barley kernels produced in the examined area resulted to be naturally contaminated by a wide range of mycotoxigenic fungi, including *Fusarium* species, which changed their relative incidence in relation to climatic conditions occurring during the seasons. The FHB complex was composed of many different *Fusarium* species but *F. poae*, *F. avenaceum*, *F. tricinctum* and *F. graminearum* resulted to be the most prevalent ones. Some species were associated with a specific barley variety and/or with specific weather parameters, indicating that the interaction between a certain plant genotype and climatic conditions may influence the presence of *Fusarium* spp. causing head infections. The FHB disease complexity makes the selection of less susceptible varieties difficult. However, the accuracy of genotype selection and the choice of cultivation areas characterized by unfavorable climatic conditions to *Fusarium* spp. infections are two parameters that play a primary role to limit the risk of FHB and mycotoxin contamination of malting barley kernels.

## 5. Materials and Methods

### 5.1. Barley Sampling, Weather Data and Anthesis Time Collection

The study was conducted on kernels of the 11 malting barley varieties Belgravia, Concerto, Esterel, Grace, Prague, Propino, Quench, Scarelett, Sunshine, Violetta and Wintmalt (Table S4) grown during the years 2010–2011, 2011–2012 and 2012–2013 in 1.5 × 6 m field experimental plots arranged in a randomized block design and located at the Experimental Station of the Department of Agricultural, Food and Environmental Sciences of the University of Perugia (Papiano, Umbria, Italy, 42°57′ N, 12°22′ E, 165 m a.s.l.). Previous crop was soft wheat (*Triticum aestivum* L.) for all three experimental years. No fungicides were applied during the three years of investigation. The varieties were sown in late autumn (Table S5) and immediately after harvest (Table S5), kernels of four field replicates of each variety were sampled. For each sample, 500 g of kernels were collected and divided into two bulks of 250 g each, one for seed mycological analyses and one, finely ground by a laboratory blender, for mycotoxin analysis. All materials were stored at 4 °C until subsequent analyses.

Weather data (average, minimum and maximum temperatures, rainfall and relative humidity) were recorded daily by a weather station at the above-mentioned Experimental Station. Anthesis time was also recorded every year for each variety and the average anthesis time was calculated for each year (Table S5). The number of days with summer temperatures above 30 and 35 °C and with winter temperatures below 0 °C were also calculated (Table 1).

### 5.2. Mycological Seed Analysis and Obtainment of Fusarium Strains

Mycological analyses of barley kernels were performed using the method described by Covarelli et al. [20]. After 5 days of incubation at 22 °C, a combination of visual and stereomicroscope observations was carried out on each kernel to morphologically identify the different fungal genera (*Fusarium*, *Alternaria*, *Aspergillus*, *Penicillium*, and *Epicoccum*) infecting the kernels and to define the fungal community composition present in the collected samples. Twenty-five seeds for each field replicate were examined for each variety for a total of 100 analyzed kernels per variety. With regard to fungal isolates possibly belonging to the *Fusarium* genus, a representative subset of colonies (about two per each replicate, when present) were transferred into new plates containing Potato Dextrose Agar (PDA, Biolife Italiana, Milan, Italy). After three weeks of incubation at 22 °C, monoconidial cultures were obtained following the method illustrated by Balmas et al. [62]. After ten days of incubation at 22 °C, pure cultures of *Fusarium* spp. were ready for DNA extraction.

### 5.3. DNA Extraction, Species Identification and Chemotype Characterization

A small piece of fungal mycelium was scraped from the surface of each colony by a micropipette tip and placed into a 200 µL plastic PCR tube (Eppendorf, Hamburg, Germany) containing 50 µL of Extraction Solution (Sigma-Aldrich, St. Louis, MO, USA). After a slight homogenization for 20 s with a micropipette tip, samples were placed into a PCR thermalcycler (Biorad, Hercules, CA, USA) at 99 °C per 10 min and then centrifuged at 12,470× *g* for 3 min on a 1–4 Sigma-Aldrich centrifuge. An amount of 50 µL of Dilution Solution (Sigma-Aldrich) was added to the centrifuged samples and 20 µL of the supernatant was transferred into a new tube and diluted to obtain a DNA concentration of ~30 ng/µL. The DNA was stored at ×20 °C until required.

*Fusarium* spp. isolated from barley kernels were identified using specific primer pairs for six *Fusarium* spp. that may potentially infect barley heads in the surveyed area. Information about all the primers used in this study is summarized in Table S6 [63,64,65,66]. A single PCR protocol was optimized using a total reaction volume of 15 µL containing 10% of 10X PCR buffer (Microtech, Pozzuoli, Naples, Italy), 0.04 ng/µL of cresol red (Sigma-Aldrich), 1.5 mM of MgCl_2_ (Microtech), 0.2 mM DNTP mix (Microtech), 0.4 μM forward and reverse primers, 0.02 U/μL of Taq polymerase (Microtech) and 2 μL of template DNA. The PCR cycle consisted of an initial denaturation step at 94 °C for 2 min followed by 35 cycles of denaturation (95 °C for 35 s), annealing (30 s at the specific annealing temperatures listed in Table S6) and extension (72 °C for 30 s), and a final extension at 72 °C for 5 min. The isolates that were not identified by species-specific PCR were subject to *TEF1α* [67,68] gene sequencing, performed by Macrogen Europe (Amsterdam, The Netherlands) and to a subsequent FUSARIUM-ID search for identification [69].

After species identification, strains identified as *F. graminearum* were characterized by multiplex-PCR [70] in a chemotype-specific test previously validated by Ward et al. [71] based on the polymorphism of the *TRI* gene cluster, which is involved in trichothecene biosynthesis, using the primers indicated in Table S6. Multiplex-PCR assays were performed in the presence of 0.4 µM of each forward primer and 0.12 µM of reverse primer (Table S6). In this case the PCR cycle had an initial denaturation step of 95 °C for 2 s followed by 30 cycles of denaturation (95 °C for 10 s), annealing (53 °C for 30 s) and extension (72 °C for 40 s) and final extension at 72 °C for 5 min. Each PCR assay contained a positive DNA control (template DNA of the target species), a negative DNA control (template DNA of a non-target species) and a negative control (no DNA added). All PCR products were loaded on a 2% TAE agarose gel in the presence of a line loaded with 1000–100 bp HyperLadder IV (Bioline, London, UK) to compare the sizes of the amplified fragments which were separated by electrophoresis applying a tension of 110 V for ~40 min. Electrophoretic runs were visualized using an UV Image analyzer (Euroclone, Pero, Milan, Italy). Specific PCR assays for *F. graminearum* were performed using a primer pair, which produces polymorphic products using DNA from *F. graminearum* lineage 7 [63]. Strains identified as *F. equiseti* (Corda) Sacc. by species-specific PCR were included in the FIESC [72].

### 5.4. Deoxynivalenol and T-2 Toxin Analysis

Mycotoxins determination was performed using the AgraQuant^®^ ELISA kits Deoxynivalenol Assay 0.25/5.0, AgraQuant^®^ T-2 toxin Assay 75/500 and AgraQuant^®^ T-2 toxin Assay 25/500 (Romer Labs^®^, USA). DON and T-2 toxin standard solutions used for the construction of the calibration curve were of 0, 250, 1000, 2000 and 5000 µg/kg for DON and 0, 75, 150, 300 and 500 µg/kg for T-2 toxin, all included in the kits. Mycotoxin extraction and determination were performed according to manufacturer’s protocols. Extraction was carried out using deionized water for DON and using methanol:water (70:30 *v*/*v*) for T-2 toxin. The optical densities of the samples and of standard curves were estimated by a multichannel photometer Multiskan EX (Labsystems, Abbiategrasso, Milan, Italy) using a 450 nm filter. A calibration curve of the standards of each toxin dilution as well as of the concentration of each mycotoxin in each sample were automatically calculated by the Romer^®^ Log/Logit spreadsheet provided by Romer^®^ Labs. The linearity coefficients (r^2^) for DON were 0.995, 0.999, 0.997 and for T-2 toxin 0.998, 0.996, 0.995. Limits of detection (LOD) of the used kits were 200 µg/kg for DON, 35 µg/kg for T-2 (2011 samples) and 15 μg/kg for T-2 (2012 and 2013 samples). Limits of quantification (LOQ) of the used kits were 250 µg/kg for DON, 75 μg/kg for T-2 toxin (2011 samples) and 25 μg/kg for T-2 toxin (2012 and 2013 samples).

### 5.5. Statistical Analysis

#### 5.5.1. Presence of Fungal Microorganisms in Malting Barley Grains in Relation to Climatic Conditions

The percentage incidence of the different fungal genera isolated from the malting varieties in the three years was submitted to ANOVA, following angular transformation to meet the basic assumptions for linear models. Variety, years and “variety × year” interaction were regarded as fixed effects. Following ANOVA, multivariate analyses were used to graphically summarize the results. In order to elucidate the effect of weather conditions on fungal incidence, the two-way matrix relating to the percentage incidence of the different fungal genera in the three years, averaged across varieties, was submitted to Principal Component Analysis (PCA), by using the function rda() of the “vegan” package in the R statistical environment [73,74]. Data were not standardized prior to analyses and results were displayed on a distance biplot (scaling 1) [75]. Environmental vectors were a posteriori projected into the ordination space, by using the function envfit() of the “vegan” package, in order to give a picture of the directions of most rapid change in the environmental variables. By using these directions, it is possible to obtain a graphical representation of the relationship between years, fungi and environmental variables, as detailed in Table 1. In order to better elucidate the relationship between genotypes and fungal genera, the two-way matrix of mean percentage incidence of the *Fusarium* genus, as observed for the three years and 11 malting barley varieties, was submitted to AMMI analysis and results were reported on an AMMI-1 biplot [76].

#### 5.5.2. Identification of *Fusarium* spp. and Their Association with Malting Barley Varieties 

Due to high number of zeros, data relating to the observed species of *Fusarium* were analyzed by non-parametric methods. In particular, the total counts of isolates observed for each species in the three years and the total counts of isolates observed for each species in the 11 barley varieties were displayed in two contingency tables and chi-square tests for independence were performed to test the significance of the variety and year effects. In order to elucidate the effects of weather pattern, these contingency tables were used to derive: (1) the percentage incidence of *Fusarium* spp. in the three years, averaged across varieties; and (2) the percentage incidence of *Fusarium* spp. in the 11 malting barley varieties, averaged across years. Both matrices were submitted to PCA as detailed above and environmental vectors were a posteriori projected into the ordination space, in order to give a picture of the directions of most rapid change in the environmental variables.

#### 5.5.3. Occurrence of Deoxynivalenol and T-2 toxin in the Grain 

DON and T2 toxin concentrations observed in the 11 varieties in the three years were submitted to ANOVA. In order to elucidate the effect of varieties, the two-way matrix of concentrations (averaged across years) classified by varieties (along the rows) and toxins (along the columns) was submitted to PCA, as detailed above.

## Figures and Tables

**Table 1:** Climatic data recorded during the three experimental years.

Time	Variable ^a^	2011	2012	2013
**Anthesis (May)**	TminA (°C)	8.6	8.1	10.3
	TavgA (°C)	17.4	15.7	15.5
	TmaxA (°C)	25.3	23.0	21.7
	RH (%)	65.0	74.2	82.3
	RainA (mm)	0.9	2.3	3.5
**Winter (December–February)**	TminAvgW (°C)	1.0	−1.1	0.7
	TminAbsW (°C)	−8.3	−12.4	−5.3
	Tbelow0 (n.)	41	55	42
**Summer (June–August) ^b^**	TmaxS (°C)	30.5	30.8	33.4
	TavgS (°C )	22.6	22.8	24.5
	Tabove25 (n.)	79	78	86
	Tabove30 (n.)	53	31	49
	Tabove35 (n.)	13	4	7

^a^ TavgA and TavgS: average temperature; TminA: minimum temperature; TmaxA and TmaxS: maximum temperature; RH: relative humidity; RainA: Rainfall; TminAvgW: average of minimum temperature; TminAbsW: absolute minimum temperature; Tbelow0: numbers of days with temperature below zero; Tabove25: numbers of days with temperature above 25 °C; Tabove30: numbers of days with temperature above 30 °C; Tabove35: numbers of days with temperature above 35 °C; ^b^ Relative to the year preceding the cultivation season.

**Table 2:** Total counts of isolates observed for the different *Fusarium* species in the three years.

Year	*F. poae*	*F. avenaceum*	*F. tricinctum*	*F. graminearum*	*F. culmorum*	FIESC ^a^	*F. proliferatum*
2011	33	1	0	6	0	6	0
2012	1	6	12	4	0	0	10
2013	23	26	0	8	1	1	0

^a^ *Fusarium incarnatum-equiseti* species complex.

**Table 3:** Deoxynivalenol (DON) and T-2 toxin grain contamination (µg/kg) in 2011, 2012 and 2013.

Varieties	DON ^b^						T-2 Toxin ^c^					
	2011		2012		2013		2011		2012		2013	
	µg/kg	±SE ^d^	µg/kg	±SE	µg/kg	±SE	μg/kg	±SE	μg/kg	±SE	μg/kg	±SE
**Belgravia**	<LOQ ^a^	-	380.9	60.2	<LOQ	-	100.3	31.8	423.7	1.2	144.3	56.4
**Concerto**	<LOQ	-	480.5	40.3	<LOQ	-	<LOQ	-	115	4.9	87.7	11.8
**Esterel**	<LOQ	-	370.1	60.6	<LOQ	-	<LOQ	-	<LOQ	-	54.1	0.9
**Grace**	270.5	30.5	470.2	70.2	<LOQ	-	244.5	198.1	<LOQ	-	108.1	11.1
**Prague**	<LOQ	-	300.2	20.5	700.2	60.2	133.3	91.2	199	0.7	100.7	17.4
**Propino**	<LOQ	-	370.3	60.6	500.5	40.4	147.8	7.4	63.3	8.2	163.4	29.9
**Quench**	280.4	10.4	440	60.2	460.6	20.2	125.4	44.1	196.8	3.6	115.4	17.7
**Scarlett**	<LOQ	-	300.2	20.4	410.3	20.7	<LOQ	-	114.1	6.9	157.6	24.4
**Sunshine**	280.7	40.3	320.8	20.3	320.4	40.3	285.9	123.6	117.4	0.3	62.7	8
**Violetta**	300.7	20.3	400.3	90.6	440.5	20.6	<LOQ	-	<LOQ	-	104.3	23.4
**Wintmalt**	250.2	60.4	340.7	50.5	610.7	10.2	<LOQ	-	162.2	6.9	236.1	61.6
**Positive (%)**	45		100		63		54		73		100	
**Average (µg/kg)**	125.6	43.7	379.4	19.1	313.0	80.5	94.3	31.5	126.5	37.2	121.3	15.6
**Range (µg/kg)**	<LOQ–300.7		300.2–480.5		<LOQ–700		<LOQ–285.9		<LOQ–423.7		54.1–236.1	

^a^ Limit of quantification; ^b^ LOQ 250 µg/kg; ^c^ LOQ 75 μg/kg (2011) and 25 μg/kg (2012 and 2013); ^d^ Standard Error.

## Supplementary Materials

The following are available online at www.mdpi.com/2072-6651/9/4/120/s1, Table S1: Incidence (%) of the different fungal genera isolated from the malting barley varieties in the three experimental years, Table S2: *F. graminearum* chemotypes in 2011, 2012 and 2013, Table S3: Total counts of isolates observed for the different *Fusarium* species in the 11 malting barley varieties, Table S4: Characteristics of the malting barley varieties analyzed in this study, Table S5: Sowing, anthesis and harvesting dates of malting barley in the three experimental years, Table S6: Primer sequences, product sizes and annealing temperatures used for PCR identification of *Fusarium* species and chemotype characterization.
