# Supplementary material for: Changes in the Fusarium Head Blight Complex of Malting Barley in a Three-Year Field Experiment in Italy
Source: Toxins (Basel). 2017 Mar 29;9(4):120. doi: 10.3390/toxins9040120 (PMC5408194; doi:10.3390/toxins9040120)
Supplement: Supplementary file 1 [file toxins-09-00120-s001.pdf]

# Supplementary Materials: Changes in the *Fusarium* Head Blight complex of malting barley in a three-year field experiment in Italy

**Giovanni Beccari, Antonio Prodi, Francesco Tini, Umberto Bonciarelli, Andrea Onofri, Souheib Oueslati, Marwa Limayma and Lorenzo Covarelli**

**Table S1.** Incidence (%) of the different fungal genera isolated from the malting varieties in the three experimental years.

| Variety | *Fusarium* | | | | | | *Aspergillus* | | | | | | *Penicillum* | | | | | | *Epicoccum* | | | | | *Alternaria* | | | | |
|---|---|---|---|---|---|---|---|---|---|---|---|---|---|---|---|---|---|---|---|---|---|---|---|---|---|---|---|---|
| | 2011 | | 2012 | | 2013 | | 2011 | | 2012 | | 2013 | | 2011 | | 2012 | | 2013 | | 2011 | 2012 | | 2013 | | 2011 [a] | 2012 | | 2013 | |
| | % | ±SE [b] | % | ±SE | % | ±SE | % | ±SE | % | ±SE | % | ±SE | % | ±SE | % | ±SE | % | ±SE | % | % | ±SE | % | ±SE | % | % | ±SE | % | ±SE |
| Belgravia | 32 | 3.5 | 25 | 1.5 | 20 | 2.4 | 11 | 1 | 26 | 0.8 | 3 | 0.7 | 4 | 1.2 | 11 | 1.3 | 2 | 0.5 | 0 | 3 | 0 | 2 | 0.5 | 0 | 29 | 4.5 | 57 | 2.7 |
| Concerto | 33 | 1.9 | 12 | 1.2 | 19 | 1.9 | 9 | 1.5 | 29 | 2.5 | 0 | 0 | 14 | 2.4 | 16 | 1.2 | 1 | 0.4 | 0 | 2 | 0.6 | 2 | 0.5 | 0 | 23 | 1 | 75 | 1.8 |
| Esterel | 32 | 2.2 | 18 | 2.1 | 22 | 1.7 | 2 | 1.6 | 13 | 1.3 | 1 | 0.4 | 9 | 1 | 24 | 4.3 | 0 | 0 | 0 | 0 | 0 | 6 | 1.4 | 0 | 41 | 5.9 | 60 | 2.2 |
| Grace | 33 | 1.6 | 13 | 1.5 | 15 | 2.1 | 5 | 0.6 | 24 | 2.2 | 1 | 0.7 | 11 | 1 | 7 | 1.3 | 0 | 0 | 0 | 3 | 1.5 | 12 | 1.8 | 0 | 37 | 1.3 | 60 | 3.2 |
| Prague | 31 | 1.7 | 15 | 1.3 | 10 | 2.1 | 15 | 0.5 | 25 | 2.6 | 0 | 0 | 1 | 0.8 | 16 | 1.2 | 1 | 0.4 | 0 | 4 | 1.2 | 5 | 1.7 | 0 | 20 | 1.4 | 56 | 2.4 |
| Propino | 37 | 2.2 | 22 | 1.7 | 18 | 2.4 | 2 | 1 | 50 | 9.8 | 2 | 1.1 | 16 | 0.1 | 18 | 1.7 | 0 | 0 | 0 | 0 | 0 | 9 | 1.1 | 0 | 28 | 3.4 | 66 | 4.1 |
| Quench | 31 | 2.2 | 21 | 1.9 | 14 | 1.6 | 7 | 1.3 | 31 | 3.7 | 0 | 0 | 6 | 1.9 | 20 | 1.4 | 2 | 0.5 | 0 | 1 | 0.5 | 6 | 1.2 | 0 | 29 | 2.5 | 60 | 2.2 |
| Scarlett | 33 | 2.5 | 29 | 1.7 | 19 | 2.1 | 5 | 1.3 | 28 | 2.8 | 1 | 0.7 | 12 | 1.8 | 17 | 3.9 | 1 | 0.5 | 0 | 1 | 0.5 | 3 | 0.7 | 0 | 34 | 1 | 63 | 2.1 |
| Sunshine | 41 | 2.2 | 28 | 2.6 | 27 | 2.5 | 5 | 1 | 20 | 0.8 | 2 | 0.7 | 12 | 2.2 | 27 | 0.5 | 1 | 0.4 | 0 | 0 | 0 | 6 | 1.6 | 0 | 26 | 1.3 | 66 | 3.3 |
| Violetta | 47 | 2.9 | 18 | 1 | 20 | 2.6 | 19 | 1.3 | 16 | 2.2 | 2 | 1.1 | 6 | 1.5 | 11 | 1.5 | 6 | 1.8 | 0 | 0 | 0 | 7 | 0.9 | 0 | 34 | 1.7 | 51 | 3.7 |
| Wintmalt | 40 | 2.1 | 17 | 3.3 | 13 | 1.3 | 18 | 0.6 | 43 | 5.7 | 2 | 0.7 | 11 | 0.5 | 22 | 2.9 | 0 | 0 | 0 | 3 | 1.5 | 5 | 1.2 | 0 | 17 | 3.4 | 55 | 3.5 |
| **Average** | 35.5 | | 19.8 | | 17.9 | | 8.9 | | 27.7 | | 1.3 | | 9.3 | | 17.2 | | 1.3 | | 0 | 1.5 | | 5.7 | | 0 | 28.9 | | 60.8 | |

| **Significance of effects** [c] | *Fusarium* | *Aspergillus* | *Penicillum* | *Epicoccum* | *Alternaria* |
|---|---|---|---|---|---|
| Year | 2E-16 | 2E-16 | 2E-16 | 0.000044 | 2E-16 |
| Variety | 0.0048 | 0.105 | 0.447 | 0.0032 | 0.078 |
| Year × Variety | 0.047 | 0.000024 | 0.000024 | 0.23 | 0.013 |

[a] In 2011 *Alternaria* incidence was not observed; [b] Standard Error; [c] *P*-levels from F tests in ANOVA

**Table S2.** *F. graminearum* chemotypes in 2011, 2012 and 2013.

| Year | Chemotypes (number of *F. graminearum* strains) | | |
|---|---|---|---|
| | NIV | 15ADON | 3ADON |
| **2011** | 0 | 6 | 0 |
| **2012** | 4 | 0 | 0 |
| **2013** | 0 | 6 | 2 |

**Table S3.** Total counts of isolates observed for the different *Fusarium* species in the 11 malting barley varieties.

| Variety | *F. poae* | *F. avenaceum* | *F. tricinctum* | *F. graminearum* | *F. culmorum* | FIESC [a] | *F. proliferatum* |
|---|---|---|---|---|---|---|---|
| Belgravia | 5 | 0 | 0 | 1 | 0 | 0 | 0 |
| Concerto | 6 | 2 | 0 | 2 | 0 | 1 | 0 |
| Esterel | 0 | 5 | 0 | 3 | 0 | 0 | 0 |
| Grace | 3 | 1 | 0 | 0 | 0 | 1 | 0 |
| Prague | 5 | 3 | 0 | 0 | 0 | 2 | 0 |
| Propino | 6 | 3 | 4 | 1 | 1 | 0 | 0 |
| Quench | 15 | 4 | 0 | 6 | 0 | 1 | 10 |
| Scarlett | 4 | 3 | 0 | 0 | 0 | 0 | 0 |
| Sunshine | 0 | 4 | 0 | 1 | 0 | 2 | 0 |
| Violetta | 6 | 3 | 8 | 4 | 0 | 0 | 0 |
| Wintmalt | 7 | 5 | 0 | 0 | 0 | 0 | 0 |

[a] *Fusarium incarnatum-equiseti* species complex

**Table S4.** Characteristics of the malting barley varieties analyzed in this study.

| Variety | Row | Type of grain | Habitus | Height of vegetation | Resistance to lodging | Resistance to diseases | | Country of origin | Main destination |
|---|---|---|---|---|---|---|---|---|---|
| | | | | | | *Puccinia hordei* | *Rhynchosporium secalis* | | |
| Belgravia | Two | Covered | Spring | High | Medium | Medium | High | UK | Malting |
| Concerto | Two | Covered | Spring | Medium | High | Medium | Medium | UK-Spain | Malting |
| Esterel | Six | Covered | Winter | High | Low | Low | High | France | Malting/feed |
| Grace | Two | Covered | Spring | Low | Medium | Medium | Medium | Germany | Malting |
| Prague | Two | Covered | Spring | Low | High | Medium | Medium | UK | Malting |
| Propino | Two | Covered | Spring | Medium | Medium | Low | Low | UK | Malting |
| Quench | Two | Covered | Spring | Medium | High | Medium | Medium | UK | Malting |
| Scarlett | Two | Covered | Spring | Medium | High | Low | Low | Germany | Malting |
| Sunshine | Two | Covered | Spring | High | Medium | Medium | Medium | Germany | Malting |
| Violetta | Two | Covered | Winter | High | Medium | Low | High | Germany | Malting |
| Wintmalt | Two | Covered | Winter | Low | Low | Medium | High | Germany | Malting |

**Table S5.** Sowing, anthesis and harvesting dates of malting barley in the three experimental years.

| Year | Sowing | Anthesis | Harvest |
|---|---|---|---|
| **2011** | 10/12/2010 | From 1/5 to 31/5/2011 | 22/6/2011 |
| **2012** | 22/12/2011 | From 1/5 to 31/5/2012 | 27/6/2012 |
| **2013** | 13/12/2012 | From 10/5 to 9/6/2013 | 17/7/2013 |

**Table S6.** Primer sequences, product sizes and annealing temperatures used for PCR identification of *Fusarium* species and chemotype characterization.

| Species/ Gene target | Primers | Sequences (5′-3′) | Product size (bp) | Annealing temp. (°C) | Reference |
|---|---|---|---|---|---|
| *F. graminearum* | Fg16F<br>Fg16R | CTCCGGATATGTTGCGTCAA<br>GGTAGGTATCCGACATGGCAA | 420 | 54 | [1] |
| *F. avenaceum* | FaF<br>FaR | CAAGCATTGTCGCCACTCTC<br>GTTTGGCTCTACCGGGACTG | 920 | 63 | [2] |
| *F. culmorum* | Fc51F<br>Fc51R | ATGGTGAACTCGTCGTGGC<br>CCCTTCTTACGCCAATCTCG | 570 | 54 | [1] |
| *F. poae* | FpsF<br>FpoR | CGCACGTATAGATGGACAAG<br>CAGCGCACCCCTCAGAGC | 400 | 61 | [3] |
| *F. equiseti* | FeqF<br>FeqR | GGCCTGCCCGATGCGTC<br>CGATACTGAAACCGACCTC | 990 | 66 | [3] |
| *F. sporotrichioides* | FsporF<br>LansporR | CGCACAACGCAAACTCATC<br>TACAAGAAGAGCGTGGCGATAT | 332 | 66 | [4] |
| *Translation Elongation Factor 1-alpha* | EF1<br>EF2 | ATGGGTAAGGA(A/G)GACAAGAC<br>GGA(G/A)GTACCAGT(G/C)ATCATGTT | 700 | 53 | [5,6] |
| *TRI12* (NIV) | 12NF(F) | TCTCCTCGTTGTATCTGG | 840 | 53 | [7] |
| *TRI12* (15ADON) | 12-15F(F) | TACAGCGGTCGCAACTTC | 670 | | |
| *TRI12* (3ADON) | 12-3F(F) | CTTTGGCAAGCCCGTGCA | 410 | | |
| *TRI12* | 12CON(R) | CATGAGCATGGTGATGTC | - | | |

## References


1. Nicholson, P.; Simpson, D.R.; Weston, G.; Rezanoor, H.N.; Lees, A.K.; Parry, D.W.; Joyce, D. Detection and quantification of *Fusarium culmorum* and *Fusarium graminearum* in cereals using PCR assays. *Physiol. Mol. Plant Pathol.* **1998**, *53*, 17–37.
2. Doohan, F.M.; Parry, D.W.; Jenkinson, P.; Nicholson, P. The use of species specific PCR based assays to analyse *Fusarium* ear blight of wheat. *Plant Pathol*. **1998**, *47*, 197–205.
3. Jurado, M.; Vazquez, C.; Patino, B.; Gonzalez-Jaen, M.T. PCR detection assays for the trichothecene-producing species *Fusarium graminearum*, *Fusarium culmorum*, *Fusarium poae*, *Fusarium equiseti* and *Fusarium sporotrichioides*. *Syst. Appl. Microbiol.* **2005**, *28*, 562–568.
4. Wilson, A.; Simpson, D.; Chandler, E.; Jennings, P.; Nicholson, P. Development of PCR assays for the detection and differentiation of *Fusarium sporotrichioides* and *Fusarium langsethiae*. *FEMS Microbiol. Lett.* **2004**, *233*, 69–76.
5. O'Donnell, K.; Kistler, H.C.; Cigelnik, E.; Ploetz, R.C. Multiple evolutionary origins of the fungus causing Panama disease of banana: Concordant evidence from nuclear and mitochondrial gene genealogies. *Proc. Natl. Acad. Sci. USA* **1998**, *95*, 2044–2049.
6. Geiser, D.M.; Jimenez-Gasco, M.D.; Kang, S.C.; Makalowska, I.; Veeraraghavan, N.; Ward, T.J.; Zhang, N.; Kuldau, G.A.; O'Donnell, K. FUSARIUM-ID v. 1.0: A DNA sequence database for identifying *Fusarium*. *Eur. J. Plant Pathol.* **2004**, *110*, 473–479.
7. Ward, T.J.; Bielawski, J.P.; Kistler, H.C.; Sullivan, E.; O'Donnell, K. Ancestral polymorphism and adaptive evolution in the trichothecene mycotoxin gene cluster of phytopathogenic *Fusarium*. *Proc. Nat. Acad. Sci. USA* **2002**, *99*, 9278–9283.
